# Features of transbronchial lung cryobiopsy‐diagnosed fibrotic hypersensitivity pneumonitis

**DOI:** 10.1111/crj.13561

**Published:** 2022-11-17

**Authors:** Xi Zhan, Yingzhen Du, Jiang Luo, Yifan Que, Chao Hu, Lili Xu, Zhen Wang, Yanbing Wu, Mulan Jin, Chunming Zheng, Yanhong Gao, Christopher Chang, Hongxia Li, Zhaohui Tong, Guogang Xu

**Affiliations:** ^1^ Department of Respiratory Medicine and Critical Care, Institute of Respiratory Medicine Beijing, Chaoyang Hospital Capital Medical University Beijing China; ^2^ Department of Respiratory and Critical Care Medicine, The Second Medical Center & National Clinical Research Center for Geriatric Diseases, Chinese PLA General Hospital Medical School of Chinese PLA Beijing China; ^3^ The Second Medical Center & National Clinical Research Center for Geriatric Diseases, Chinese PLA General Hospital Medical School of Chinese PLA Beijing China; ^4^ Department of Pathology, Chaoyang Hospital Capital Medical University Beijing China; ^5^ Medical Research Center, Chaoyang Hospital Capital Medical University Beijing China; ^6^ Division of Pediatric Immunology, Allergy and Rheumatology Joe DiMaggio Children's Hospital Hollywood Florida USA; ^7^ Division of Rheumatology, Allergy and Clinical Immunology University of California, Davis Davis California USA

**Keywords:** fibrotic hypersensitivity pneumonitis, interstitial lung diseases, pulmonary fibrosis, transbronchial lung cryobiopsy, usual interstitial pneumonia

## Abstract

**Background:**

Hypersensitivity pneumonitis (HP) is a common type among all the interstitial lung diseases, and transbronchial lung cryobiopsy is an alternative diagnostic technique for interstitial lung diseases. In this study, we describe the clinical and pathological features of fibrotic hypersensitivity pneumonitis diagnosed with transbronchial lung cryobiopsy (TBLC).

**Methods:**

A total of 46 diffused parenchyma lung disease (DPLD) patients received TBLC were included in this study. Medical records including medical history spirometry examinations, 6‐min walk test (6MWT) results, high resolution computed tomographic (HRCT) scans, BAL, and histopathology were collected. Results of HRCT and histopathology were compared and classified, especially.

**Results:**

Sixteen patients were diagnosed with fibrotic HP, the mean age of whom was 56.3 ± 12.1 years, and 62.5% of them were male. Three of the 16 patients had been misdiagnosed as tuberculosis and received antituberculosis medications, five patients had been diagnosed as unclassifiable pulmonary fibrosis, and five patients had been diagnosed as idiopathic pulmonary fibrosis (IPF). Thirteen (81.3%) patients had a normal lymphocyte count in BAL. The pathological features of usual interstitial pneumonia (UIP) were detected in 11 (68.8%) of the cases, poor defined granulomatous was detected in nine (56.3%) of the cases, and bronchiolocentric fibrosis was detected in two (12.5%) of the 16 cases.

**Conclusions:**

Fibrotic hypersensitivity pneumonitis should be included in differential diagnosis of pulmonary fibrosis. Pathological characteristics of fibrotic hypersensitivity pneumonitis could be demonstrated from cryobiopsy lung tissue. TBLC is recommended as an alternative diagnostic technique, which may improve the specificity of hypersensitivity pneumonia detection, and UIP is the most frequent pathological finding.

## Graphical abstract

This study describes the clinical and pathological features of fibrotic hypersensitivity pneumonitis diagnosed with transbronchial lung crybiopsy. Usual interstitial pneumonia was the most frequent pathological finding of fibrotic hypersensitivity pneumonitis. Transbronchial lung crybiopsy could be considered as a diagnostic technique for fibrotic hypersensitivity pneumonitis.

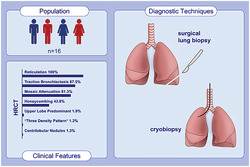

## 1 INTRODUCTION

Hypersensitivity pneumonitis (HP), or extrinsic allergic alveolitis, is an interstitial lung disease (ILD). A Brazilian cohort comprising 3168 ILD cases showed that HP accounted for 15% of all ILDs, ranking second only to connected tissue disease‐related ILD (CTD‐ILD, 17%).^1^ The worldwide incidence of HP was reported therefore, HP is more often clinically defined as acute or chronic, based on disease duration (lessor more than 6 months).^2^ The acute and chronic subtypes appear to be more consistent with nonfibrotic and fibrotic HP, respectively. However, there is no strict relation between symptom duration and fibrotic status.^3^

The mechanism of progression of HP to the fibrotic stage is still unclear. The type of “inducer” influences the pattern of HP; for instance, antigens contained in bird feathers and droppings seem to induce fibroproliferative responses.^4^ In patients with farmer's lung disease, which often presents as combined fibrosis and emphysema syndrome, HP may be a consequence of chronic inflammation.^5^

The clinical presentation of HP is remarkably diverse. History of exposure to organic or inorganic antigens, upper lobe predominance and mosaic attenuation on CT, and increased lymphocytes in bronchoalveolar lavage (BAL) are clues for diagnosis. However, a stimulating antigen is not found in as many as 50–60% of HP patients.^3^ Moreover, lower lung predominant fibrosis is identified in 30–50% of subjects. Furthermore, some cases of fibrotic HP cannot be distinguished from IPF^6^ due to the absence of a BAL lymphocyte increase.^1^ Upper‐zone predominant fibrosis has been considered a characteristic feature for distinguishing fibrotic HP from IPF. However, ≤10% of fibrotic HP patients were reported to have the upper‐lung‐predominant pattern.^7^, ^8^ In some cases, doctors may be unable to confirm a diagnosis of HP; thus, lung biopsy and a multiple disciplinary treatment (MDT) team are required for the diagnosis.

Transbronchial lung cryobiopsy (TBLC), an emerging relatively safe and easy operative technique, is an effective approach that can help to establish a diagnosis of HP. TBLC provides lung samples with much fewer complications than those of traditional surgical lung biopsies (SLBs).^9^ The utilization of TBLC in ILD centers is increasing; however, its diagnostic accuracy compared with that of SLB is still controversial. This is because the only two prospective multicenter studies of TBLC reached different conclusions.^10^ In this study, we aim to clarify the clinical, HRCT, and pathological features of fibrotic HP based on TBLC and explore the possible mechanism of inhaled substances causing HP.

## 2 MATERIAL AND METHODS

### 2.1 Study design and participants

HRCT was used to diagnose HP where meshwork, pullable branching, or honeycomb on HRCT were diagnostic for fibrosis.^3^ Patients with no conclusive diagnosis after MDT experts discussion received TBLC. In total, 46 DPLD patients who were admitted to Chaoyang Hospital and underwent TBLC from June 2017 to June 2020 were enrolled. All diagnosis were made by MDT team discussions. The MDT team was composed of two pulmonologists, one rheumatologist, two occupational physicians, one pathologist, and two radiologists. Rounds of discussion were necessary if there was disagreement to assure a final consensus. Patients were followed up regularly to obtain survival data.

### 2.2 Data collection

Patient information was extracted from electronic medical records. The information collected included medical history, smoking history, exposure history, co‐morbidities, spirometry examinations, 6‐min walk test (6MWT) results, high resolution computed tomographic (HRCT) scans, BAL, and histopathology.

### 2.3 TBLC procedure

Targeted locations were chosen according to the following imaging features: ① reticular pattern or traction bronchiectasis/bronchiolectasis mixed with mosaic attenuation, ② reticular pattern or traction bronchiectasis/bronchiolectasis with infiltration, and ③ no honeycombing.

Prior to TBLC, the patients were anesthetized with intravenous injection of propofol and then intubated through rigid bronchoscopes. Continuous monitoring of ECG, blood pressure, and pulse oxygen saturation in patients were conducted during the entire operation. Cryoprobes were placed in the working channel of the bronchoscope to the targeted location, which was identified by HRCT prior to the procedure. The probe was then cooled down to −80°C for 3 to 7 s. The cryoprobe, with attached frozen lung tissue, was removed together with the flexible bronchoscope. The mean lung sample diameter was 5 mm. Routinely, two to five samples were collected per patient. The frozen biopsy tissues were then thawed with saline buffer and placed in a 10% formalin solution. Prophylactic endobronchial balloon blockers were used to stop any bleeding after TBLCs. The biopsies were analyzed by experienced pathologists at the Pathology Department of Chaoyang Hospital.

### 2.4 Statistical analysis

The clinical data of all patients enrolled in the study were described and analyzed. These included age, gender, smoking history, exposure history, pulmonary function values (spirometry data, 6MWT, PaO_2_ of arterial blood gas), HRCT, and histopathology. Continuous data are presented as numbers with percentages (%). BAL lymphocytes were assessed regardless of being within the normal range or not. IBM SPSS Statistics for Windows, version 26.0 (IBM Corp., Armonk, N.Y., USA), was used for all statistical analyses.

## 3 RESULTS

### 3.1 Presenting characteristics

**FIGURE 1:**
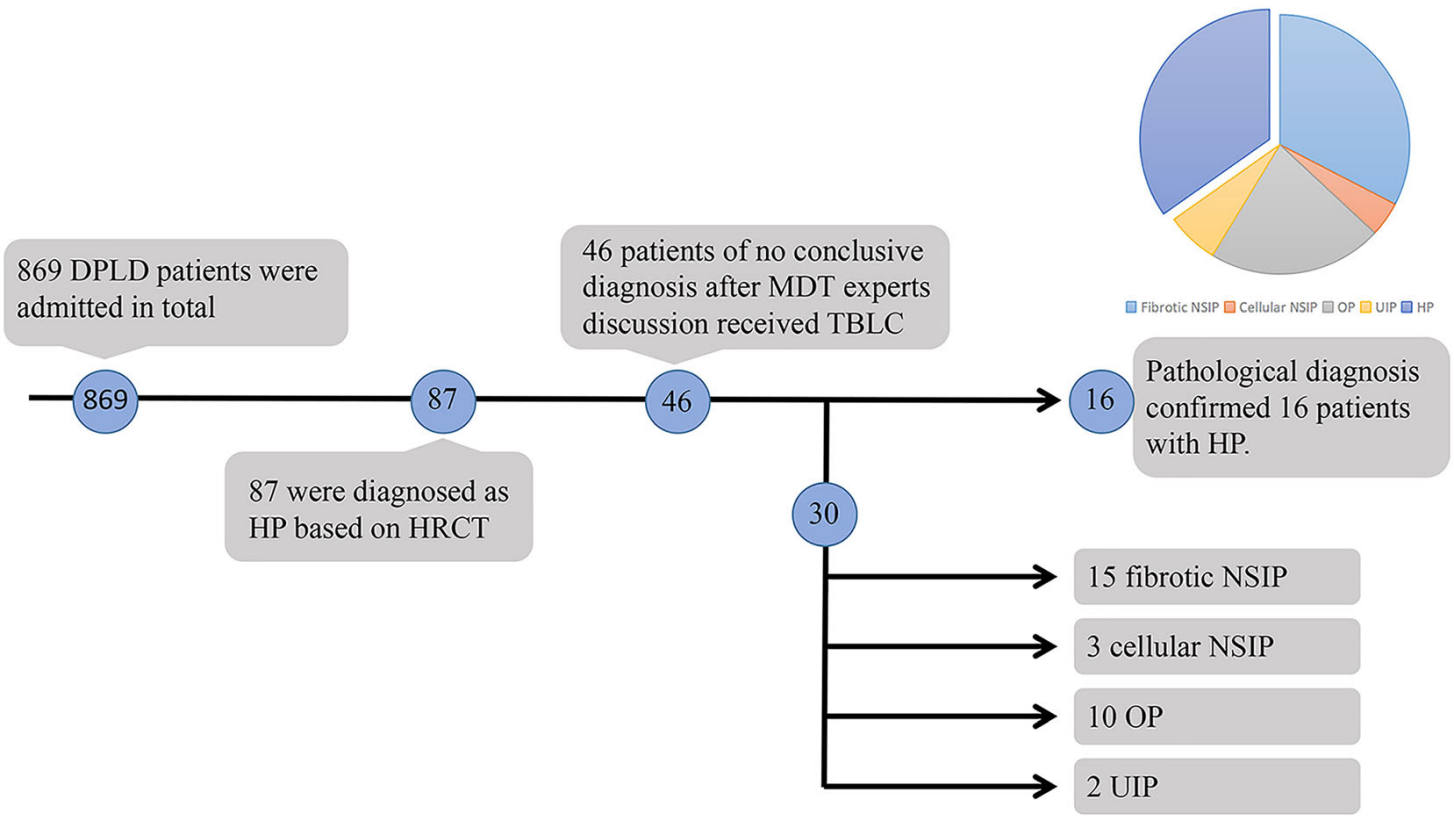
Patient flowchart. DPLD, diffused parenchyma lung disease; HRCT, high‐resolution computed tomography; MDT, multi‐discipline treatment; TBLC, transbronchial lung cryobiopsy; NSIP, nonspecific interstitial pneumonia; OP, organizing pneumonia; UIP, usual interstitial pneumonia; HP, hypersensitivity pneumonitis

Among 869 patients with DPLD who were admitted to hospital in our study, 87 were diagnosed with HP based on HRCT. Only 46 patients who had inconclusive diagnoses after MDT experts' discussions underwent TBLC. After TBLC, 30 patients were diagnosed with different diseases based on pathological results: 15 fibrotic and three cellular nonspecific interstitial pneumonia (NSIP), 10 organizing pneumonia (OP), and two usual interstitial pneumonia (UIP). Figure 1 shows a flowchart detailing patient flow in our study.

Sixteen (40%) patients were diagnosed with fibrotic HP according to the 2020 HP guideline.^3^ Table 1 shows the baseline data of the 16 patients. All the 16 patients had not received TBLB during previous conventional bronchoscopy. None of the 46 patients receiving TBLC underwent surgical lung biopsy for further diagnosis. Before admission in our hospital, three, five, and five of the 16 patients had been misdiagnosed with tuberculosis (received antituberculosis medications), unclassifiable pulmonary fibrosis, and IPF, respectively, in other hospitals. Four patients had a history of feeding birds, three had been living in a moist environment with mold on the walls of their living rooms, one had a history of exposure to molded grain for years, and no history of exposure was discovered in the remaining eight patients. No drug/recreational drug exposure history was discovered. All 16 patients had negative autoimmune series laboratory test results, and those with connective tissue diseases would have been excluded. Nine patients had inspiratory squawk on auscultation. None of the 16 patients received steroids, immunosuppressants, or antifibrotic therapy before TBLC. Regular bronchoscopy was applied for all the 16 cases, for BAL before TBLC.

**TABLE 1:** Baseline data

Characteristic	Mean ± SD/N (%)
Gender	
Female	6 (37.5%)
Male	10 (62.5%)
Age (years)	
Average Age	56.3 ± 12.1
Range	45–67
Smoking history	
Never smoked	4 (25%)
Ex‐smoker	1 (6.25%)
Current smoker	11 (68.75%)
Exposure history	
Birds	4 (25%)
Moisture	3 (18.75%)
Molded grain	1 (6.25%)
Co‐morbidity	
Gastroesophageal reflux	5 (31.25%)
Congenital dyskeratosis	1 (6.25%)

*Notes*: Data are shown as mean ± standard deviation (SD); count values and percentages are presented in brackets.

Among the 16 cases diagnosed as chronic fibrotic HP, the lesion distribution was as follows: Eight patients were sampled at the left lower lobe (six and two at the lateral and posterior basal segments, respectively). Right lower lobe biopsies included five and three at the lateral and posterior basal segments, respectively.

**FIGURE 2:**
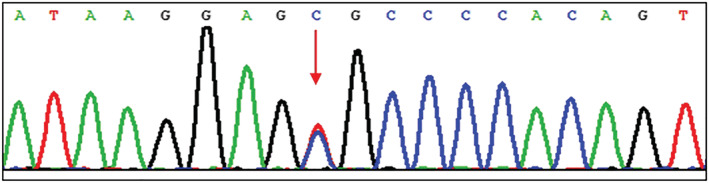
Whole exome sequence for the congenital dyskeratosis patient showed a nonsense mutation (c.844C>T) in *TINF2* exon 14.

The mean age was 56.3 ± 12.1 years, and 62.5% of the patients (10/16) were male. Eleven of the patients were current smokers, one was an ex‐smoker, and four had no smoking history. Five patients had co‐morbidities of gastroesophageal reflux, confirmed by a 24‐h gastroesophageal pH and motility monitor. One patient had a co‐morbidity of congenital dyskeratosis (Table 1), with a nonsense mutation (c.844C>T) in TINF2 exon 14 (Figure 2). The mutation was detected by whole exome sequencing.

### 3.2 HRCT imaging findings

**FIGURE 3:**
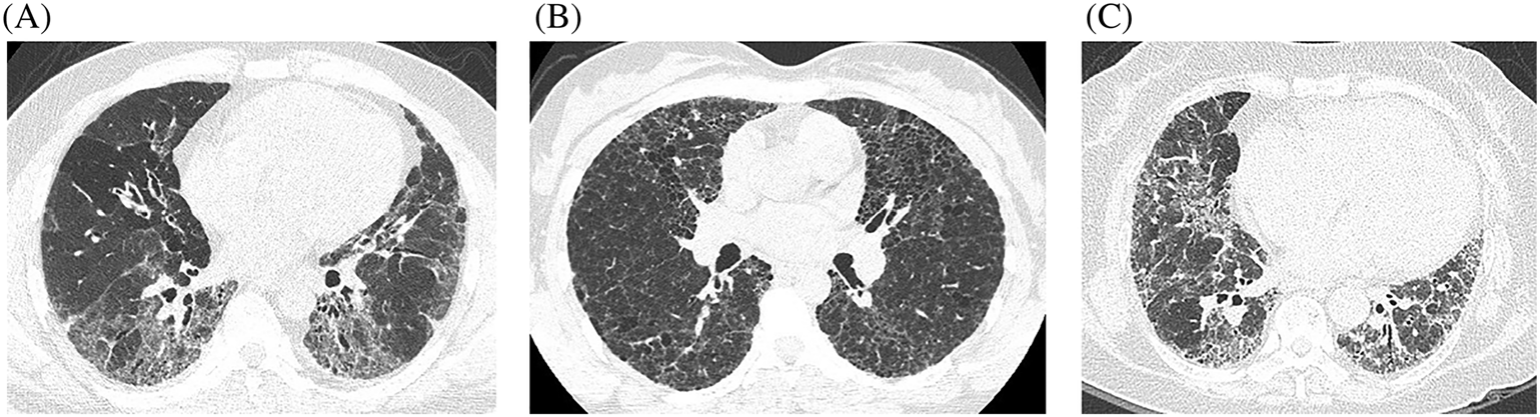
HRCT results in patients with fibrotic HP. (A) A male patient with fibrotic HP. HRCT showed reticulation, traction bronchiectasis, and mosaic attenuation. (B) A female patient with fibrotic HP. HRCT showed reticulation and honeycombing. (C) A female patient with chronic fibrotic HP. HRCT showed reticulation and a “three density pattern” (headcheese sign). HRCT, high‐resolution computed tomography; HP, hypersensitivity pneumonitis

The two radiologists read all the HRCT scans together, and there was consensus on interpretation. Of the cases, 18.8% (3/16) had upper lobe‐predominant disease, 100% had a reticular fibrotic pattern (Figure 3A,B), 43.8% (7/16) had honeycombing (Figure 3B), mosaic attenuation was found in 81.3% (13/16) (Figure 3A), and the “three density pattern” (headcheese sign) was found in 12.5% (2/16) (Figure 3C).

### 3.3 Laboratory and pathological findings

**FIGURE 4:**
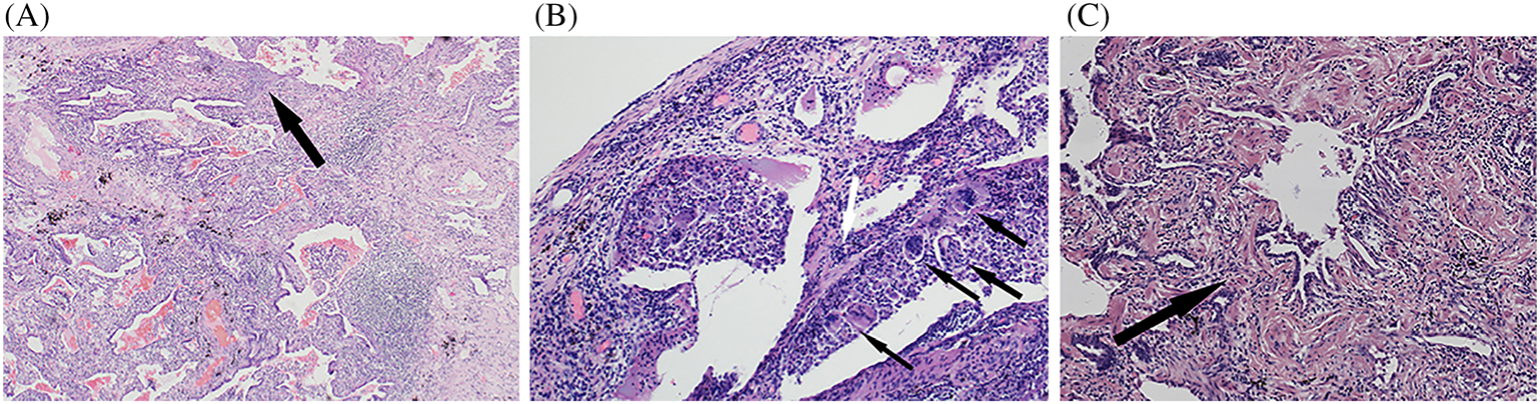
Pathological features in patients with fibrotic HP. (A) A female patient with fibrotic HP. The pathology of the TBLC sample showed patchy fibrosis and fibroblast foci, in accordance with UIP. (Black Arrow HE 40 Χ). (B) A male patient with fibrotic HP. The pathology of the TBLC sample showed poor defined granulomas (black arrows) distributed along with small airways, and fibroblast foci (white arrow). (HE 100 Χ). (C) A male patient with fibrotic HP. The pathology of the TBLC sample showed bronchiolocentric fibrosis (black arrow, HE 100 Χ). TBLC, transbronchial lung cryobiopsy; HP, hypersensitivity pneumonitis

Spirometry tests demonstrated a mean forced vital capacity (FVC) of 72.3 ± 8.1% of predicted value. The mean diffused capacity (DLCO) was 60.8 ± 9.7% of predicted value; 81.3% (13/16) had a normal BAL lymphocyte count. Eleven of the 16 cases displayed UIP‐like pathological features (Figure 4A). The FVC and BAL lymphocyte count had no significant differences between the pathological UIP group (*n* = 11) and non‐UIP group (*n* = 5); the *P* value was 0.18 and 0.33, respectively. The differences of DLCO between groups was not calculated because of its measurement variability. Poorly defined granulomatous and bronchiolocentric fibrosis were detected in 56.3% (Figure 4B) and 12.5% (Figure 4C), respectively, of the 16 cases. All the pathological slides were interpreted by the same pathologist. A UIP‐like pattern was characterized by subpleural or paraseptal fibrosis alternating with normal alveoli, temporal heterogeneity, and structural distortion. Table 2 shows the clinical features of the patients.

**TABLE 2:** Clinical features

Feature	Mean ± SD/N (%)
Course of disease (months)	11.7 ± 3.5
Inspiratory squawk	9 (56.3%)
PaO_2_ (mmHg) of arterial blood gas (room air)	73.1 ± 5.7
Spirometry	
FVC (%predicted value)	72.3 ± 8.1
DLCO (%predicted value)	60.8 ± 9.7
6‐Minute walk test (room air)	
Distance (meters)	342 ± 56.7
Nadir SpO_2_	89 ± 5.1
HRCT	
Upper lobe predominant	3 (18.8%)
Reticulation	16 (100%)
Traction bronchiectasis	14 (87.5%)
Honeycombing	7 (43.8%)
Mosaic attenuation	13 (81.3%)
“Three density pattern”	2 (12.5%)
Centrilobular nodules	2 (12.5%)
BAL	
Lymphocytes >15%	13 (81.3%)
Lymphocytes <15%	3 (18.8%)
Histopathology	
Poorly defined granulomas	9 (56.3%)
Bronchiolocentric fibrosis	2 (12.5%)
Usual interstitial pneumonia	11(68.8%)

*Notes*: Data are presented as mean ± standard deviation (SD); count values and percentages are presented in brackets.

Abbreviations: BAL, bronchoalveolar lavage; DCLO, diffusing capacity of lungs for carbon monoxide; FVC, forced vital capacity; HRCT, high‐resolution computed tomography.

## 4 DISCUSSION

The diagnosis of fibrotic HP remains challenging. Serum specific IgG is helpful; however, the diagnostic kits are not available in China. The specific inhalation challenge test (bronchial challenge test, BCT) for diagnosis was reported to have a sensitivity and specificity of 72.7% and 84%, respectively. However, a negative test result did not rule out HP. Moreover, BCT was poorly standardized and not widely available. Consequently, its use was limited to patients with severely impaired lung function.^11^, ^12^

Hundreds of antigens have been confirmed to cause HP, and questionnaires of exposure history have been used when collecting history.^4^, ^13^ However, despite thorough elicitation for exposure history, the “inducer” is still unknown in many confirmed HP cases. Therefore, diagnosis has to be made using histopathological findings, as well as typical clinical and radiological features.

The radiological features of fibrotic HP include reticulation; traction bronchiectasis, with or without honeycombing; mosaic attenuation; and the “three density pattern,” all of which are suggestive of air trapping.^3^ The term “three density pattern” was introduced in the 2020 HP guideline. It emphasizes that attenuated and vascularized lobules on inspiratory HRCT images are of diagnostic value. This is especially true when exhalation is accompanied by air retention, which is highly correlated with severe bronchiolar obstruction.^3^

Interlobular septal thickening can be profuse in fibrotic HP. An upper lobe predominant distribution is often seen. However, diffuse and lower lobe predominant changes have also been described in 31% of chronic HP cases. Further, in the same study, lower zone predominance was found in 83% of IPF cases.^14^ Slight bronchiolocentric fibrosis can be observed in the lung apices, and reticulation is demonstrated to have a predominantly subpleural or peribronchovascular distribution. In a Japanese cohort of 31 patients with chronic HP, a reticular pattern and traction bronchiectasis were found in 100% (all) and 84% (26/31) of cases, respectively. These results did not differ significantly with those of the IPF group. However, honeycombing was seen in 29% (9/29) of cases, which was much lower than that seen in IPF cases (100%). The mosaic pattern, ground glass opacity, and centrilobular nodules were more common in the chronic HP group than in the IPF group.^15^

All the cases (100%) in our cohort presented as a reticular pattern; 87.5% (14/16) had traction bronchiectasis, and 43.75% (7/16) had honeycombing on HRCT scans. Of our cohort, 18.75% (3/16) who had been previously misdiagnosed with tuberculosis had an upper lobe predominant distribution. Hints of air trapping could be seen in lung focal areas with no increase in attenuation. This manifests as mosaic attenuation on HRCT scans, which is one of the characteristic features of chronic HP but is not exclusive to HP. Significant air trapping can be visualized as mosaic attenuation on HRCT inspiratory imaging.^16^ In our cohort, mosaic attenuation was found in 81.25% (13/16) of cases in the inspiratory phase and the “three density pattern” was detected in 12.5% (2/16) of cases.

Histopathology in fibrotic HP is characterized by chronic bronchiolitis, with patchy fibrosis, fibroblastic foci, and occasional poorly formed granulomas. Poorly formed granulomas are identified in about 50–58% cases of chronic HP and most are seen in the peribronchiolar area.^17^ Giant cells are commonly seen in granulomas, and cholesterol clefts are found in their cytoplasm.^17^ In our study, ill‐defined granulomas were seen in 56.3% of cases (9/16), which was consistent with the SLB findings of other studies.^17^ Takemura et al. studied pathological DPLD features differentiating HP with a UIP‐like pattern from IPF based on SLB histology.^17^ They defined chronic HP with a UIP‐like pattern as centrilobular fibrosis, bridging fibrosis, organizing pneumonia, bronchiolitis, granulomas, and giant cells. However, in IPF patients, granulomas and giant cells were rarely seen in the fibrotic areas.^17^ An international modified Delphi survey demonstrated that air trapping, mosaic attenuation on HRCT scans, and poorly formed lung biopsy non‐necrotizing granulomas could be the featured radiological and pathological characteristics for fibrotic HP.^13^ In our study, the UIP‐like pattern and bronchiolocentric fibrosis were seen in 68.8% and only 12.5%, respectively, of the 16 cases. In Takemura's study, centrilobular fibrosis was seen in 100% of the chronic HP cases.^17^ The difference may be due to the relatively small sample size of cryobiopsy in both studies.

Inspiratory squawk, a high‐pitched end‐inspiratory wheeze (accompanied by rales or not), is a relatively specific sign of fibrotic HP.^18^ Inspiratory squawk was detected in 56.3% (9/16) of our patients. Bronchiolocentric inflammation is considered the earliest lesion of HP,^19^ and inspiratory squawk is a sign of bronchiolitis. Inspiratory squawk can also be heard in patients with bronchiolitis obliterans associated with rheumatoid disease.^18^ In cases of DPLD with inspiratory squawk, fibrotic HP may be a diagnostic consideration.

Gastroesophageal reflux is not direct evidence of aspiration. This is due to methodologic flaws in the testing of gastric biomarkers in airways. The test results are important in assessing how gastroesophageal reflux affects respiratory disorders. Gastroesophageal reflux is a risk factor for chronic bronchoaspiration, which causes a variety of pulmonary disorders, including pulmonary fibrosis.^20^, ^21^ Instead of gastroesophageal reflux, histopathologic appearance of foreign bodies with well‐formed granulomatous is the best evidence to prove how aspiration causes respiratory diseases. Foreign bodies with well‐formed granulomatous were not found in the specimens of the five cases with gastroesophageal reflux; therefore, chronic bronchoaspiration‐related pulmonary fibrosis was excluded by the MDT.

The mechanism of the fibrotic process of HP is not fully elucidated. HP is characterized by Th1 cell immunity and immune complex‐mediated lung injury, which may involve specific IgG antibodies.^4^, ^22^ However, in the later stages of HP, the Th1 response switches to a Th2 response, leading to persistent inflammation and development of fibrosis.^22^ The Th17 immune response, in parallel with the Th1 response, may be activated by overproduction of IL‐17. Interleukin 17 is a powerful stimulator of chronic inflammation.^23^ A fibroproliferative response may be attributed to neutrophils and fibrocytes through their humoral paracrine effect.^23^ Smoking is thought to reduce the risk of HP; however, HP patients with a smoking history sometimes present with a chronic fibroproliferative course. This is caused by an increase in the Th2 response resulting from a decreased activity of Th1 and Th17 lineages.^24^

The average age of our cohort was 56.3 ± 12.1 years, and 75% (12/16) of the patients had a smoking history. Some fibrotic HP patients had telomere shortening related fibrosis.^25^ One patient in our cohort had a comorbidity of congenital dyskeratosis, with a nonsense mutation (c.844C>T) in *TINF2* exon 14 (Figure 2). The mutation correlated with telomerase malfunction, leading to telomere length shortening.

The prognosis of chronic HP depends on the pathological pattern. Wang's study of 119 patients with chronic HP, diagnosed using SLB pathological findings, showed transplant‐free survival with the fibrotic NSIP pattern. The prognosis of patients with a bronchiolocentric fibrosis (BF) pattern, or UIP, were worse than those of patients with cellular NSIP or peribronchiolar inflammation with poorly formed granulomas (PI‐PFG) patterns. In addition, fibroblastic foci, identified by biopsy in 30.3% of cases, were associated with progression to poor prognoses, such as lung transplantation.^26^ Fibroblastic foci were found in 25% (4/16) of cases in our cohort, and survival data are being collected during follow‐up.

Transbronchial cryobiopsy has been used in many ILD centers as it allows larger sample sizes and higher diagnostic yields than those of conventional transbronchial biopsies. Moreover, TBLC has a lower complication rate than that of SLB. A comparison of pooled diagnostic yields between cryobiopsy (83.7%) and video‐assisted thoracoscopic SLB (92.7%) showed a slightly lower cryobiopsy accuracy.^27^ Furthermore, the incidences of significant bleeding and pneumothorax post‐transbronchial cryobiopsy were considered acceptable (4.9% and 9.5%, respectively).^27^ In our cohort of HP patients, significant bleeding and pneumothorax rates were 6.25% (1/16) and 12.5% (2/16), respectively. Cryobiopsy is not recommended by the 2018 IPF guideline for newly diagnosed ILD patients with unknown causes, such as those with a UIP HRCT pattern. For patients with newly identified ILD whose differential diagnosis includes fibrotic HP, the committee suggests TBLC.6 However, due to the lack of prospective studies, no recommendation was made either for or against cryobiopsy for patients with a probable UIP, indeterminate UIP in HRCT, or other diseases.^28^ The two prospective studies, published in 2019,^10^, ^29^ comparing the concordance between cryobiopsy and SLB reached different conclusions.

The limitations of our study were obvious. The study was retrospective with a small sample size. Further research is needed to establish the specific diagnostic yield of cryobiopsy in relation to distinguishing fibrotic HP from IPF.

## 5 CONCLUSIONS

To conclude, our cohort showed that pathological characteristics of fibrotic HP could be demonstrated from cryobiopsy lung tissue. Our study recommends TBLC as an alternative diagnostic technique, which may improve the specificity of hypersensitivity pneumonia detection and draw attention on the study of UIP.

## CONFLICT OF INTEREST

The authors confirm that there are no competing interests.

## AUTHOR CONTRIBUTIONS

**Xi Zhan:** Writing–Original Draft Preparation; Data Curation; Formal Analysis; Writing–Review & Editing. **Yingzhen Du:** Writing–Original Draft Preparation; Writing–Review & Editing. **Jiang Luo:** Writing–Original Draft Preparation; Writing–Review & Editing; **Yifan Que:** Writing–Original Draft Preparation; Writing–Review & Editing. **Chao Hu:** Investigation; Methodology. **Lili Xu:** Investigation; Methodology. **Zhen Wang:** Investigation; Methodology. **Yanbing Wu:** Investigation; Methodology. **Mulan Jin:** Methodology; Resources; Validation. **Chunming Zheng:** Software; Supervision. **Yanhong Gao:** Formal Analysis; Writing–Review & Editing. **Hongxia Li:** Conceptualization; Writing–Review & Editing. **Christopher Chang:** Writing–Review & Editing. **Zhaohui Tong:** Conceptualization; Writing–Review & Editing. **Guogang Xu:** Conceptualization; Writing–Review & Editing.

## ETHICS STATEMENT

This retrospective cohort study was performed in Chaoyang Hospital. The study protocol was officially approved by the Chaoyang Hospital Ethics Committee (No. 2020‐253) and conformed to the Declaration of Helsinki. According to the Ethics Committee, our study is retrospective research; therefore, patient consent statements have been granted an exemption although each patient had signed the standard consent statement before TBLC.

## Data Availability

The data that support the findings of this study are available from the corresponding author upon reasonable request.
